# Primary Human Dendritic Cells and Whole-Blood Based Assays to Evaluate Immuno-Modulatory Properties of Heat-Killed Commensal Bacteria

**DOI:** 10.3390/vaccines9030225

**Published:** 2021-03-05

**Authors:** James E. Norton, Sushma Kommineni, Patricia Akrivoulis, Dario A. Gutierrez, Daria J. Hazuda, Gokul Swaminathan

**Affiliations:** Exploratory Science Center, MRL, Merck & Co., Inc., Cambridge, MA 02141, USA; james.norton@merck.com (J.E.N.J.); sushma.kommineni@merck.com (S.K.); Patricia.Akrivoulis@novartis.com (P.A.); dario.gutierrez@tcr2.com (D.A.G.); daria.hazuda@merck.com (D.J.H.)

**Keywords:** immune modulation, host–microbiome interactions, commensal bacteria, microbiome, dendritic cells, innate immunity

## Abstract

There is mounting evidence that the microbiome plays a critical role in training and maturation of the host immune system. Pre-clinical and clinical studies have shown that microbiome perturbation is correlated with sub-optimal host responses to vaccines and cancer immunotherapy. As such, identifying species of commensal bacteria capable of modulating immunological outcomes is of considerable interest. Currently, the lack of reliable primary immune cell-based assays capable of differentiating immuno-modulatory properties of various commensal bacteria is a major limitation. Here, we demonstrate that primary human monocyte-derived dendritic cells (MoDC) are capable of stratifying different strains of live and heat-killed commensal bacteria in an in vitro culture system. Specifically, heat-killed bacterial strains were able to differentially modulate co-stimulation/maturation markers CD80, CD83, and HLA-DR, as well as cytokine/chemokine signatures, such as IL-1b, MIP-1a, and TNFa in primary human MoDC. We further validated our observations using the TruCulture^®^ (Myriad RBM, Inc., Austin, TX, USA) whole-blood ex vivo culture system. Using this ex vivo system allowed us to measure immune-altering effects of commensal bacteria in primary human whole-blood. As such, we report that both these primary in vitro and ex vivo systems are robust and enable identification, stratification, and differentiation of various commensal bacteria as potential modulators of host immunity.

## 1. Introduction

Mounting evidence has shown that the host microbiome plays a critical role in proper development, maturation, and function of the host immune system. This phenomenon has been well-characterized in germ-free murine studies, where the absence of a normal microbiome results in impaired host immune function [1,2] and in human clinical observational studies [3,4,5]. Additionally, variations in normal host microbiome composition has been linked to reduced efficacy in immune system-altering therapies, including vaccine response [6,7,8,9] and anti-PD-1 immunotherapy [10,11,12] in humans. Understanding ways in which to modulate sub-optimal immune responses is of considerable interest.

One potential way to restore immune tone is by altering or normalizing the microbiome through the administration of commensal bacteria. However, current methods used to identify and rank potential immuno-modulators often fall short in characterizing the potential immune-altering properties of commensal bacteria. Much of this kind of work relies upon immortalized and/or non-primary cell lines for screening [13]. These cell lines are often poor indicators of immune modulation in an in vitro culture system, due to biased expression of specific innate immune receptors which may contribute to the selection of false positives during the screening process. Additionally, while in vivo animal models are often used as a complete biological system, there tends to be a lack of translatability from mouse to human. Due to these pitfalls, it is likely that potential potent immuno-modulators have been either misidentified or missed entirely. Therefore, developing reliable and sensitive in vitro/ex vivo culture systems to identify and rank potential immuno-modulators of the host immune system is one of the first steps towards solving such a problem.

Monocytes and dendritic cells (DC) are both critical antigen-presenting immune cells that play important roles in human health and host immunity [14,15,16,17,18]. Monocytes also have the capacity to differentiate into DC and are more easily isolated from human whole-blood than DC [19,20]. For these reasons, we hypothesized that monocyte-derived dendritic cells (MoDC) would be a logical choice for the screening of potential immuno-modulators in a primary in vitro culture system. While primary human MoDC have been used to evaluate immuno-modulatory properties of commensal bacteria in the past [21,22], these studies were limited by the use of live bacteria only, and often confounded by co-culture with innate immune agonists or immune cells, such as NK cells or T-cells. Here, we chose a larger subset of bacterial species and strains to more thoroughly validate primary human MoDC as a sensitive cell type to measure commensal bacteria-based immuno-modulation, while also eliminating innate immune agonists and co-culturing of additional cell types that may confound endpoint readouts. When choosing commensal bacteria to use in this in vitro culture system, we chose a mixture of Gram positive (+ve) and Gram negative (-ve) commensal bacteria, with known biological and clinical importance [23,24,25,26,27]. As such, we investigated the potential use of primary human monocytes, differentiated into dendritic cells, in an in vitro culture system to identify, stratify, and rank live and heat-killed commensal bacteria as potential modulators of host immunity. Successfully establishing and validating this primary in vitro culture system will enable us to identify commensal bacteria capable of host immuno-modulation, as measured by upregulation of cell surface co-stimulation/maturation markers CD80 (B7.1), CD83, and HLA-DR, as well as secreted pro-inflammatory cytokines/chemokines, including IL-1b, MIP-1a, MIP-1b, and TNFa, amongst others.

In addition to our primary human MoDC in vitro culture system, we also investigated the potential use of the TruCulture^®^ whole-blood ex vivo culture system as a means of further identifying, stratifying, and ranking the immuno-modulatory capacity of various strains of commensal bacteria. One advantage of using the TruCulture^®^ system is that primary human whole-blood does not require any manipulation prior to use. Past published studies have shown that stimulation of human whole-blood via the TruCulture^®^ ex vivo culture system increased reproducibility and reduced background levels of cytokines for accurate interpretation of immuno-modulation with agonists, cytokines, and microbial entities, including pathogenic bacteria [28,29]. This self-contained culture system allowed us to understand potential immuno-modulation in an unmanipulated, more complete biological system. Use of the TruCulture^®^ ex vivo whole-blood culture system yielded consistent, robust measurement of secreted pro-inflammatory cytokines/chemokines, including G-CSF, IL-8, and MIP-1a, amongst others.

Taken together, our findings show that a primary human MoDC in vitro system and a primary human whole-blood ex vivo system both appear to be sensitive ways of stratifying different genera of both Gram-positive (+ve) and Gram-negative (-ve) bacteria. Additionally, both can detect strain-based differences within the same species of bacteria with high confidence. As a result, we suggest that both systems described herein be used cooperatively to positively identify live and/or heat-killed commensal bacteria that have the potential to modulate host immunity and have a beneficial impact upon sub-optimal host immune responses.

## 2. Materials and Methods

### 2.1. Growth of Commensal Bacteria

Isolates of commensal intestinal bacterial species listed in Table 1 were purchased from American Type Culture Collection (ATCC, Manassas, VA, USA). Each strain was cultured individually in media overnight (16–18 h) or for 48 h in aerobic or anaerobic conditions. Brain Heart Infusion media (BHI) or Yeast Casitone Fatty Acids with Carbohydrates (YCFAC) broth (both from Anaerobe Systems, Inc., Morgan Hill, CA, USA) was used for culturing bacteria. Bacteria were harvested by centrifugation at 4000 rpm for 15 min, washed three times and suspended in pre-reduced anaerobically sterilized (PRAS) dilution buffer (Anaerobe Systems, Inc., Morgan Hill, CA, USA). Bacteria were counted using a hemocytometer under a microscope and suspended at a concentration range of 10^7^–10^9^ cells per mL, as verified by viable counting. For heat-killed bacteria, suspensions after microscopic enumeration were subjected to heating at 121 ℃ for 30 min. Efficiency of heat killing method was verified by culturing for viability.

### 2.2. In Vitro Differentiation and Stimulation of Primary Human Monocyte-Derived Dendritic Cells (MoDC) with Live Bacteria

Primary human monocytes (CD14^+^) obtained from healthy human donors were purchased from Biological Specialty Corporation/BioIVT (Colmar, PA, USA). All donors were screened and confirmed to be negative for blood-borne pathogens (data not shown). Upon receipt, monocytes were differentiated into dendritic cells by incubation in MoDC media (RPMI-1640 with 10% heat-inactivated fetal bovine serum (FBS), 1% Penicillin/Streptomycin, 1% HEPES buffer, 50 ng/mL recombinant human GM-CSF, and 50 ng/mL recombinant human IL-4) [RPMI-1640, FBS, Penicillin/Streptomycin, HEPES buffer from ThermoFisher Scientific, Waltham, MA, USA; IL-4, GM-CSF from R&D Systems, Inc., Minneapolis, MN, USA] for three days at 37 ℃, 5% CO_2_ in T75 or T150 tissue culture flasks (Corning, Inc., Corning, NY, USA), without agitation. Following incubation, cells were washed with fresh MoDC media and transferred into new T75 or T150 tissue culture flasks for an additional three days at 37 ℃, 5% CO_2_, without agitation. Following incubation, cells were washed with MoDC media, counted, and seeded at 10^6^ cells/mL in MoDC media in 12-well tissue culture plates (Corning, Inc., Corning, NY, USA). Single-cell suspensions were then stimulated with 10^4^ cfu (live) or 10^4^ particles (heat-killed) *Fusobacterium nucleatum* (Fn23726), *Bacteroides fragilis* (Bf25285), or *Fusobacterium nucleatum* (Fn25586) in 1mL MoDC media overnight (16–18 h) at 37 ℃, 5% CO_2_, without agitation [all strains from ATCC, Manassas, VA, USA]. PBS (ThermoFisher Scientific, Waltham, MA, USA) served as a negative control. N = 3 independent donors.

### 2.3. In Vitro Differentiation and Stimulation of Primary Human Monocyte-Derived Dendritic Cells (MoDC) with Heat-Killed Bacteria

Primary human monocytes (CD14^+^) obtained from healthy human donors were purchased and differentiated into dendritic cells as described above. Differentiated MoDC were seeded at 10^6^ cells in 100 uL MoDC media in 96-well U-bottom tissue culture plates (Corning, Inc., Corning, NY, USA). Single-cell suspensions were then stimulated with 10^4^ particles of heat-killed *Bifidobacterium breve* (Bb15700), *Akkermansia muciniphila* (AmBAA-835), *Fusobacterium nucleatum* (Fn23726), *Bacteroides fragilis* (Bf25285), *Faecalibacterium prausnitzii* (Fp27766), *Enterococcus hirae* (Eh8043), *Bacteroides fragilis* (Bf43858), *Fusobacterium nucleatum* (Fn25586), or *Escherichia coli* K-12 (K12), or 1 ug/mL high molecular weight (HMW) polyinosinic:polycytidylic acid (Poly I:C) in 100 uL MoDC media overnight (16–18 h) at 37 ℃, 5% CO_2_, without agitation [*Escherichia coli* K-12 from Sigma-Aldrich, St. Louis, MO, USA; all other strains from ATCC; HMW Poly I:C from InvivoGen, San Diego, CA, USA]. PBS (ThermoFisher Scientific, Waltham, MA, USA) served as a negative control. N = 6 independent donors.

### 2.4. Quantification of Adenosine Triphosphate (ATP) and Immune Mediators in Cell Culture Supernatant

500 uL live- and heat-killed bacteria-stimulated MoDC cell culture supernatant was harvested from 12-well tissue culture plates (Corning, Inc., Corning, NY, USA), centrifuged at 400× *g* for 10 min, recovered, aliquoted into pyrogen-free tubes (Fisherbrand, Waltham, MA, USA), and stored at –80 ℃. Adenosine triphosphate (ATP) levels were measured via colorimetric ATP assay kit (abcam, Cambridge, MA, USA), as per manufacturer instructions, and read on a Synergy Neo2 Hybrid Multi-Mode Reader (BioTek Instruments, Inc., Winooski, VT, USA). Immune mediator levels in human MoDC cell culture supernatant were analyzed by multi-analyte bead-based immune assay (#HCYTMAG-60K-PX38, MilliporeSigma, Burlington, MA, USA), adapted for use with 96-well DropArray plates and DropArray LT210 washing station (both from Curiox Biosystems, Woburn, MA, USA), and read on a MAGPIX xMAP instrument (Luminex, Corp., Austin, TX, USA). N = 3–6 independent donors.

150 uL heat-killed bacteria-stimulated MoDC cell culture supernatant was harvested from 96-well U-bottom tissue culture plates after centrifugation at 400× *g* for 10 min at room temperature. Cell culture supernatant was recovered, aliquoted into a new, sterile 96-well U-bottom tissue culture plate, and stored at –80 ℃. Immune mediator levels in human MoDC cell culture supernatant were analyzed as outlined above. N = 6 independent donors.

### 2.5. Immuno-Staining and Flow Cytometry

Heat-killed bacteria-stimulated MoDC were pelleted in 96-well U-bottom tissue culture plates via centrifugation at 400× *g* for 10 min. at room temperature. Cells were washed twice with PBS and viable cells were determined by incubating in PBS with 1% zombie green viability dye (BioLegend, Inc., San Diego, CA, USA). Cells were washed twice with stain buffer (BD Biosciences, San Jose, CA, USA) and non-specific binding was blocked by incubating in stain buffer with 1% human FC block (BD Biosciences, San Jose, USA) for 20 min on ice. Cells were washed twice with stain buffer and antibody staining was performed by the addition of fluorophore-conjugated antibodies for 30 min at 4 ℃. Cellular surface markers included APC-conjugated CD80 (clone 2D10), APC-H7-conjugated HLA-DR (clone G46-6), PE-conjugated CD86 (clone FUN-1), BV421-conjugated CD197 (clone 150503), and BV786-conjugated CD83 (clone HB15e) [CD80 from BioLegend, Inc., San Diego, CA, USA; CD83, CD86, CD197, HLA-DR from BD Biosciences, San Jose, USA]. Cells were washed twice and resuspended in stain buffer prior to flow cytometry analysis. Fluorescently labeled cells were analyzed using a FACSCelesta flow cytometer (BD Biosciences, San Jose, USA) and FlowJo v.10.4.2 software (BD Biosciences, San Jose, USA). N = 6 independent donors.

### 2.6. Stimulation of Primary Human Whole-Blood with Heat-Killed Bacteria Using the TruCulture^®^ Ex Vivo Whole-Blood Culture System

TruCulture^®^ tubes (Myriad RBM, Inc., Austin, TX, USA) were purchased and loaded with 10^4^ particles of heat-killed *Bifidobacterium breve* (Bb15700), *Fusobacterium nucleatum* (Fn23726), *Fusobacterium nucleatum* (Fn25586), or 1 ug/mL high molecular weight (HMW) polyinosinic:polycytidylic acid (Poly I:C) [all strains from ATCC, Manassas, VA, USA; HMW Poly I:C from InvivoGen, San Diego, CA, USA]. TruCulture^®^ tubes that were not loaded with HK bacteria served as a negative control. Human Whole-blood was drawn into TruCulture^®^ tubes as per manufacturer instructions, tubes were inverted 3-5X, and incubated in a dry heat block for 24 h (+/− 15 min) at 37 ℃ without agitation. N = 3 independent donors.

### 2.7. Quantification of Immune Mediators in Human Sera

Sera was harvested from TruCulture^®^ tubes as per manufacturer instructions, aliquoted into pyrogen-free tubes (Eppendorf North America, Enfield, CT, USA), and stored at −80 ℃. Immune mediator levels in human sera were analyzed by multi-analyte bead-based immune assay (#HCYTMAG-60K-PX38, MilliporeSigma, Burlington, MA, USA), adapted for use with 96-well DropArray plates and DropArray LT210 washing station (both from Curiox Biosystems, Woburn, MA, USA), and read on a MAGPIX xMAP instrument (Luminex, Corp., Austin, TX, USA). N = 3 independent donors.

### 2.8. Statistical Analysis

Immuno-stained cell surface markers and quantification of immune mediators in cell culture supernatant and human sera were analyzed using an unpaired parametric t-test with Welch’s correction via GraphPad Prism 8.1.1 software (GraphPad Software, Inc., San Diego, CA, USA). For the quantification of immuno-stained cell surface markers, mean fluorescence intensity (MFI) [geometric mean] of each conjugated-fluorochrome was used. For quantification of mediators in cell culture supernatant and human sera, incalculable values below the lowest value of the standard curve (3.2 pg/mL) are represented as one half the minimum detectable concentration, whereas incalculable values above the highest value of the standard curve (10.000 pg/mL) were excluded from analysis. Data is presented as mean +/− standard error of the mean (SEM). Where appropriate, N sizes are shown in the figure legends. Where indicated in the figures, * *p* < 0.05, ** *p* < 0.01, *** *p* < 0.001, **** *p* < 0.0001.

## 3. Results

### 3.1. Primary Human MoDC Stimulated with Live and Heat-Killed Bacteria Demonstrate Comparable Levels of Immune Activation

Primary human monocytes (CD14^+^) obtained from healthy human donors were differentiated into MoDC and stimulated with 10^4^ cfu (live) or 10^4^ particles (heat-killed) of *Fusobacterium nucleatum* (Fn23726), *Fusobacterium nucleatum* (Fn25586), or *Bacteroides fragilis* (Bf43858) overnight (16–18 h) at 37 ℃, 5% CO_2_, without agitation. PBS served as a negative control. Previous experiments evaluating cellular cytotoxicity via adenosine triphosphate (ATP) release and flow cytometry analysis of cell surface co-stimulation/maturation markers allowed us to determine that 10^4^ cfu live bacteria or 10^4^ particles heat-killed (HK) bacteria was the optimal concentration to stimulate the primary human MoDC in this in vitro culture system (data not shown). Following incubation, cell culture supernatant was harvested, and secreted, soluble mediators were measured via multi-analyte bead-based immune assay.

**Figure 1:**
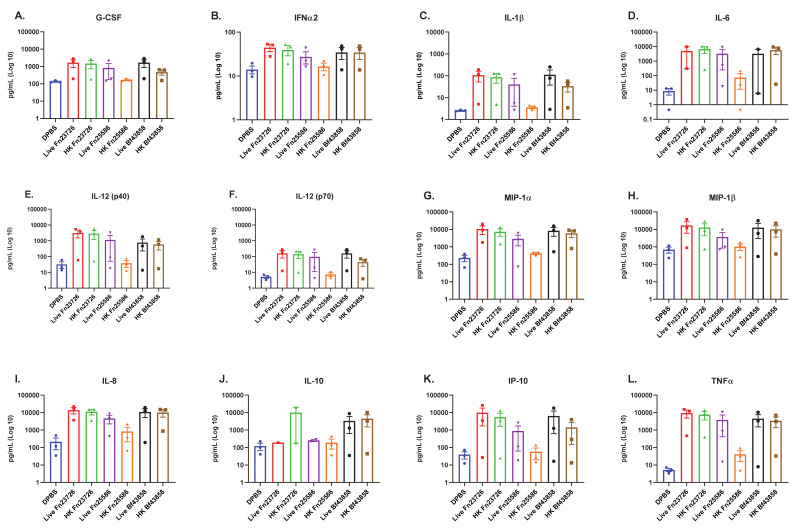
Stimulation of Primary Human MoDC Show Similar Pro-Inflammatory Mediator Profiles with Live and Heat-Killed Bacteria. Primary human MoDC were analyzed for activation, as measured by upregulation of secreted, soluble mediators in cell culture supernatant. The mean + standard error of the mean (SEM) in Log10 scale of G-CSF (**A**), IFNa2 (**B**), IL-1b (**C**), IL-6 (**D**), IL-12 (p40) [**E**], IL-12 (p70) [**F**], MIP-1a (**G**), MIP-1b (**H**), IL-8 (**I**), IL-10 (**J**), IP-10 (**K**), and TNFa (**L**) are indicated. To compare two groups of data, an unpaired parametric *t*-test with Welch’s correction was used. N = 3 independent donors.

We observed that primary human MoDC stimulated with live Fn23726, Fn25586, and Bf43858 induced the upregulation of numerous pro-inflammatory immune mediators, as seen in Figure 1. The largest upregulation of pro-inflammatory immune mediators was observed for IL-1b, IL-6, IL-12 (p40), MIP-1a, IL-8, IP-10, and TNFa, as seen in Figure 1C–E,G,I,K,L, respectively. Though upregulation was not as profound as those mentioned above, we also observed upregulation of pro-inflammatory G-CSF, IFNa2, IL-12 (p70), and MIP-1b, as seen in Figure 1A,B,F,H, respectively. There appears to be a greater level of variability in upregulation of anti-inflammatory IL-10 in Figure 1J where there is a slight upregulation in primary human MoDC stimulated with live Bf43858, though it is not significant. Most notably, primary human MoDC stimulated with live Fn23726, Fn25586, and Bf43858 exhibited very similar pro-inflammatory immuno-modulatory signatures across all three bacteria. The minor differences between live Fn23726, Fn25586, and Bf43858 can be attributed to donor-to-donor variability, since three unique donors were used in this assay. As such, all primary human MoDC from different donors showed similar immuno-modulatory signatures when stimulated with the three live bacteria used here, irrespective of donor and statistical significance.

We observed similar findings after stimulation of these primary human MoDC with HK bacteria, as well. Both HK Fn23726 and Bf43858 appear to have similar pro-inflammatory immuno-modulatory signatures whether the strains were heat-killed or used live. We again see the most profound upregulation of IL-1b, IL-6, IL-12 (p40), MIP-1a, IL-8, IP-10, and TNFa in Figure 1C–E,G,I,K,L, respectively. Consistent with live bacteria-stimulated primary human MoDC, we also observed upregulation of pro-inflammatory G-CSF, IFNa2, IL-12 (p70), and MIP-1b, as seen in Figure 1A,B,F,H, respectively. Primary human MoDC stimulated with HK Fn25586 exhibited the poorest pro-inflammatory immuno-modulatory profile of the three HK bacteria and live bacteria used here. This pattern of low expression was observed for all pro-inflammatory immune mediators assessed in Figure 1A–I,K,L, in addition to anti-inflammatory IL-10 in Figure 1J. Notably, the differences in soluble immune mediator signatures of primary human MoDC stimulated with HK Fn23726 or Fn25586 is an important finding in that this primary human MoDC in vitro culture system may be sensitive enough to detect strain-based differences in immuno-modulatory capability. Identifying these strain-based differences is critical when determining strains with which to move forward. It may be possible to observe statistically significant upregulation of immune mediator levels with an increased donor number in future studies.

Prior to moving forward, we set out to determine if this concentration of bacteria was killing the primary human MoDC used in our in vitro culture system. As such, we performed an adenosine triphosphate (ATP) release colorimetric assay to measure cell cytotoxicity, if any, as per manufacturer instructions. This quantifiable measurement of ATP is justified here since ATP is only produced by metabolically active, functional cells [30]. Figure S1 shows that ATP is produced in appreciable amounts by primary human MoDC when stimulated with live Fn23726, Fn25586, or BF25285. In contrast, ATP is produced at or below assay background levels when stimulated with HK Fn23726, Fn25586, or Bf25285. These differences are highlighted in the dashed boxes of Figure S1. The slight differences in background detection can be attributed to donor-to-donor variability inherent in the use of primary human cells. Additionally, all other HK bacteria (Bb15700, AmBAA-835, Bf43858, Fp27766, and Eh8043) used here did not stimulate the production of appreciable amounts of ATP in primary human MoDC above background. These findings indicate that our chosen concentration of live or HK bacteria is not overtly killing the primary human MoDC used in our in vitro culture system. However, given our observations in Figure 1 and Figure S1 and practical real-world considerations, such as (i) utilizing HK bacteria more readily than live bacteria and (ii) the use of live anaerobic bacteria with immune cells cultured in aerobic conditions, we chose to further evaluate only HK bacteria as our key immuno-modulatory agents moving forward.

### 3.2. Primary Human MoDC Stimulated with Heat-Killed Bacteria Can Detect Strain-Based Differences in Immuno-Modulation

All HK bacteria chosen to further validate this primary human in vitro culture system can be found in Table 1. As such, primary human monocytes (CD14^+^) obtained from healthy human donors were differentiated into MoDC and stimulated with 10^4^ particles of heat-killed (HK) *Bifidobacterium breve* (Bb15700), *Akkermansia muciniphila* (AmBAA-835), *Fusobacterium nucleatum* (Fn23726), *Bacteroides fragilis* (Bf25285), *Faecalibacterium prausnitzii* (Fp27766), *Enterococcus hirae* (Eh8043), *Bacteroides fragilis* (Bf43858), or *Fusobacterium nucleatum* (Fn25586), or 1 ug/mL HMW Poly I:C overnight (16–18 h) at 37 ℃, 5% CO_2_, without agitation. PBS served as a negative control. Following incubation, cells were harvested and immuno-stained for cell surface expression of CD80 (B7.1), CD83, CD86 (B7.2), CD197 (CCR7), and HLA-DR. Figure S2 shows a representative sample of the flow cytometry gating strategy employed for all primary human MoDC donors analyzed in this study.

**Figure 2:**
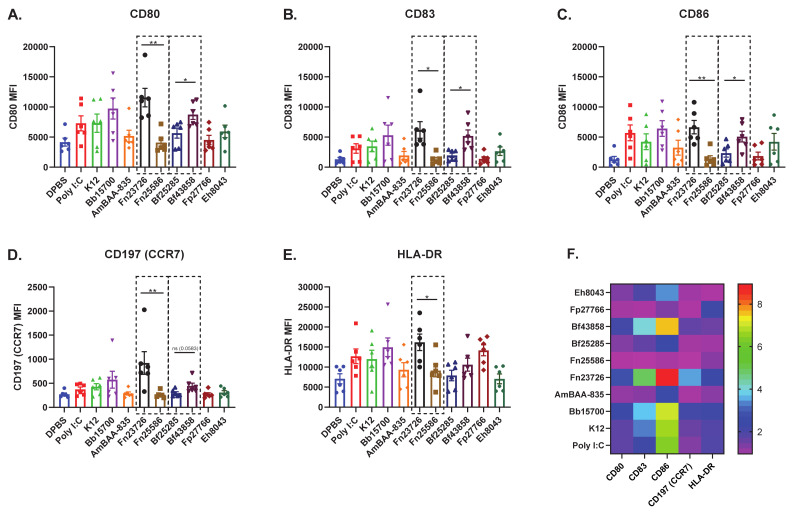
Stimulation of Primary Human MoDC Shows Upregulation of Activation Markers Which Helps Stratify Heat-Killed Bacteria and Can Differentiate Different Strains of the Same Heat-Killed Bacteria. Primary human MoDC were analyzed for activation, as measured by the upregulation of cell surface expression of CD80, CD83, CD86, CD197 (CCR7), and MHCII in heat-killed bacteria-stimulated cultures. The geometric mean fluorescence intensity (MFI) of CD80 (**A**), CD83 (**B**), CD86 (**C**), CD197 (CCR7) [**D**], and HLA-DR (**E**) are indicated. The fold increases of mean fluorescence intensity of treatment groups, calculated based on the DPBS treatment group (M), are indicated. To compare two groups of data, an unpaired parametric *t*-test with Welch’s correction was used. N = 6 independent donors. (* *p* < 0.05, ** *p* < 0.01).

As previously outlined via soluble immune mediator response signatures in Figure 1, this primary human MoDC in vitro culture systems appears sensitive enough to detect strain-based differences in immuno-modulatory capability. To further confirm this finding, we measured strain-based stimulation differences in immuno-modulation via mean fluorescence intensity (MFI) [geometric mean] of cell surface co-stimulation/maturation markers CD80, CD83, CD86, CD197 (CCR7), and HLA-DR, shown in Figure 2. As expected, well established innate agonists such as HMW Poly I:C induced a robust increase of all co-stimulation/maturation markers in primary human MoDC as compared to the DPBS control group. As another positive control, LPS was used to stimulate primary human MoDC and the data is included in Figure S3. We see variability in upregulation of CD80, CD83, CD86, CD197 (CCR7), and HLA-DR in primary human MoDC stimulated with various HK bacterial strains; for example, Bb15700>K12>Fp27766. Importantly, we also noticed that stimulation with HK Fn23726 resulted in statistically significant upregulation of all markers, compared to stimulation with Fn25586, as seen in Figure 2. To further validate the sensitivity of this assay to differentiate two separate strains of the same species of bacteria, we chose two strains of Bacteroides fragilis. HK Bf43858-stimulated primary human MoDC more effectively upregulated CD80, CD83, CD86,), and HLA-DR, compared to stimulation with Bf25285, as seen in Figure 2A–C,E, respectively. Differences between HK Fn25586 and Fn23726 and HK Bf43858 and Bf25285 are highlighted in the dashed boxes of Figure 2A–E. Although we observed minor donor-to-donor variability based on specific treatment conditions, we observed a consistent intra-donor pattern of upregulation of all co-stimulation/maturation markers measured for each treatment group.

As such, we were again able to identify strain-based differences following stimulation with different HK commensal bacteria using primary human MoDC, as in Figure 1. These consistent findings across five different cell surface markers add an additional layer of confidence that primary human MoDC are a sensitive cell type to use in this in vitro culture system to measure modulation of host immunity in a consistent, reliable, and quantifiable manner. The wider spread of values observed here was somewhat expected, given donor-to-donor variability inherent in the use of primary human cells. In an effort to highlight the sensitivity of these read-outs to differentiate between two distinct strains of the same bacteria, we chose to only show the statistics depicted in the dashed boxes. Additional statistical comparisons across the various bacterial strains tested against the negative control (DBPS) and positive control (Poly I:C) can be found in Table S1.

**Figure 3:**
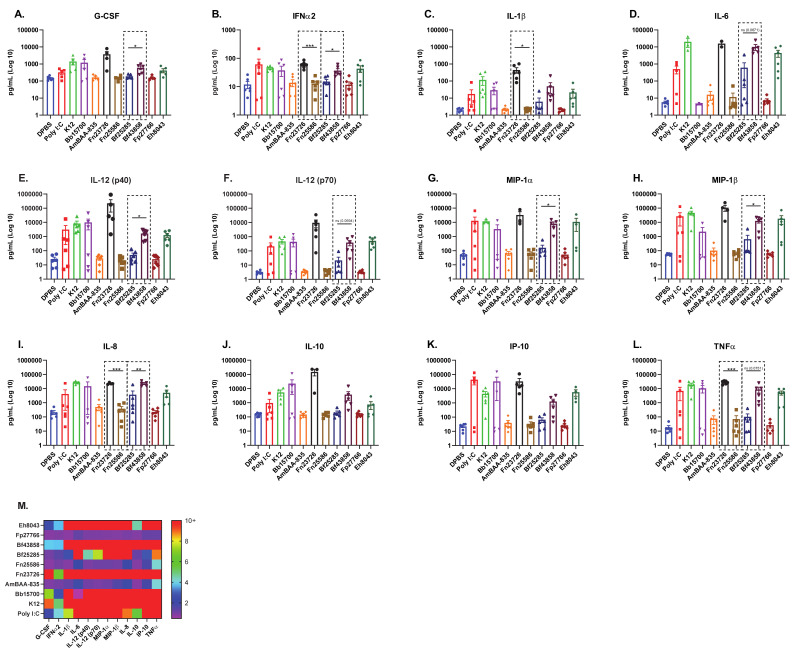
Stimulation of Primary Human MoDC Shows Upregulation of Pro-Inflammatory Mediators Which Further Stratifies and Differentiates Heat-Killed Bacteria. Primary human MoDC were analyzed for activation, as measured by upregulation of secreted, soluble mediators in cell culture supernatant. The mean + standard error of the mean (SEM) in Log10 scale of G-CSF (**A**), IFNa2 (**B**), IL-1b (**C**), IL-6 (**D**), IL-12 (p40) [**E**], IL-12 (p70) [**F**], MIP-1a (**G**), MIP-1b (**H**), IL-8 (**I**), IL-10 (**J**), IP-10 (**K**), and TNFa (**L**) are indicated. The fold increases of soluble immune mediators of treatment groups, calculated based on the DPBS treatment group (**M**), are indicated. To compare two groups of data, an unpaired parametric *t*-test with Welch’s correction was used. N = 6 independent donors. (* *p* < 0.05, ** *p* < 0.01, *** *p* < 0.001).

**Figure 4:**
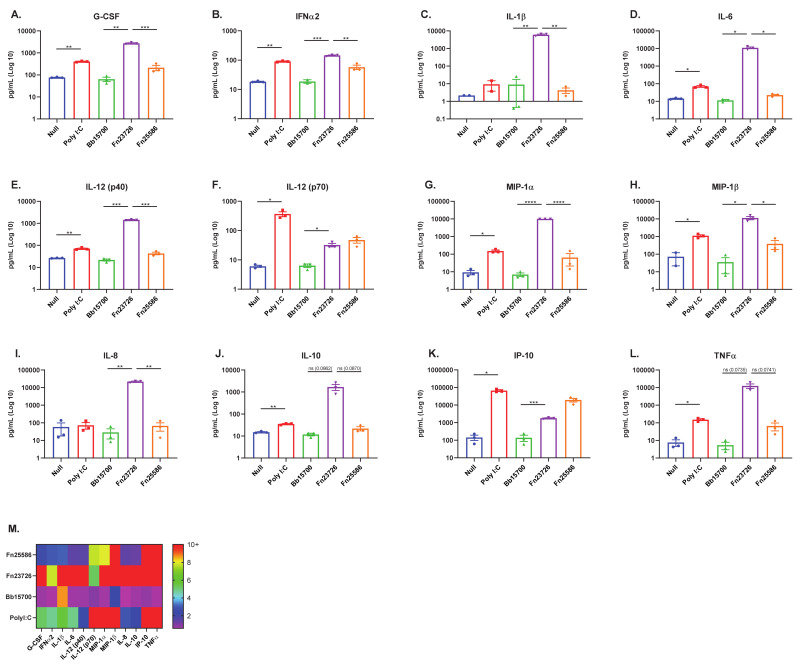
Stimulation of Primary Human Whole-Blood via TruCulture ex vivo System Further Validates Stratification and Differentiation of Heat-Killed Bacteria. Primary human whole-blood was analyzed for activation, as measured by upregulation of secreted, soluble mediators in sera. The mean + standard error or the mean (SEM) in Log10 scale of G-CSF (**A**), IFNa2 (**B**), IL-1b (**C**), IL-6 (**D**), IL-12 (p40) [**E**], IL-12 (p70) [**F**], MIP-1a (**G**), MIP-1b (**H**), IL-8 (**I**), IL-10 (**J**), IP-10 (**K**), and TNFa (**L**) are indicated. The fold increases of soluble immune mediators of treatment groups, calculated based on the Null treatment group (**M**), are indicated. To compare two groups of data, an unpaired parametric *t*-test with Welch’s correction was used. N = 3 independent donors. (* *p* < 0.05, ** *p* < 0.01, *** *p* < 0.001, **** *p* < 0.0001).

Next, we further analyzed the immuno-modulation of these primary human MoDC (N = 6) via measurement of soluble immune mediators in cell culture supernatant. Cell culture supernatant was harvested from all samples immediately prior to cell surface immuno-staining and secreted, soluble mediators were measured via multi-analyte bead-based immune assay. As seen in Figure 1 and Figure 2, primary human MoDC stimulated with HK Fn23726 again showed upregulation of key pro-inflammatory immune mediator levels above those seen with stimulation of Fn25285, shown in Figure 3. Pro-inflammatory immune mediators G-CSF, IFNa2, IL-1b, IL-6, IL-12 (p40), IL-12 (p70), MIP-1a, MIP-1b, IL-8, IP-10, and TNFa were all upregulated after stimulation with HK Fn23726 compared to Fn25286, as seen in Figure 3A–I,K–L, respectively. This is also the true with respect to anti-inflammatory IL-10 in Figure 4J. Regardless of statistical-significance, mediator levels from HK Fn23726-stimulated primary human MoDC are all elevated above levels seen in Fn25286-stimulated primary human MoDC. These data coincide nicely with what was observed in Figure 1 and Figure 2 following stimulation with HK Fn23726 and Fn25286. Additionally, we again see that HK Bf43858 is superior to Bf25285 in stimulating primary human MoDC. Pro-inflammatory immune mediators G-CSF, IFNa2, IL-1b, IL-6, IL-12 (p40), IL-12 (p70), MIP-1a, MIP-1b, IL-8, IP-10, and TNFa were all upregulated after stimulation with HK Bf43858 compared to Bf25285, as seen in Figure 3A–I,K,L, respectively, in addition to anti-inflammatory IL-10 in Figure 4J. These soluble immune meditator signatures lend an additional level of confidence to our observed findings of cell surface co-stimulation/maturation markers from Figure 2 that show better induction of immune modulation with HK Bf43858 than Bf25285. These strain-based differences in immuno-modulation are highlighted in the dashed boxes of Figure 3.

We again see a remarkably consistent pattern of pro- and anti-inflammatory immune mediators in DPBS-, HMW Poly I:C-, K12-, Bb15700-, AmBAA-835-, Fp27766-, and Eh8043-stimulated primary human MoDC, as seen in Figure 3A–L. Notably, primary human MoDC stimulation with HK Bb15700, Fn23726, and Bf43858 have similar cell surface co-stimulation/maturation marker expression patterns, while HK AmBAA-835, Fn25586, and Fp27766 exhibit similar expression patterns. HK Eh8043 most-closely resembles the expression patterns of Fn25586 and Bf25285. These expression patterns were also seen between the above listed HK bacteria in cell surface co-stimulation/maturation markers following primary human MoDC stimulation, as seen in Figure 2. This finding, along with stratification and differentiation of different strains of bacteria, further adds to our confidence in the use of primary human MoDC in this in vitro culture system. As in Figure 2, we chose to only show the statistics depicted in the dashed boxes to further highlight the sensitivity of these read-outs to differentiate between two distinct strains of the same bacterial species. However, additional statistical comparisons across the various bacterial strains tested against the negative control (DBPS) and positive control (Poly I:C) can be found in Table S2.

### 3.3. Primary Human Whole-Blood Stimulated with Heat-Killed Bacteria Can Detect Strain-Based Differences in Immuno-Modulation

Due to the practical constraints of working with human donors, we narrowed our focus to three HK bacteria for experiments using human-whole blood. To further evaluate strain-based differences, Gram -ve HK Fn23736 and Fn25586 were chosen, since *Fusobacteria* have been known to modulate the immune system [31,32]. Additionally, Gram +ve Bb15700 was chosen based on the favorable results outlined in Figure 2 and Figure 3, and the data showing *Bifidobacteria* to be a well-characterized probiotic bacterium in the human setting, with known immuno-modulatory properties [33,34]. Furthermore, selection of these bacterial strains allowed us to evaluate both Gram -ve and Gram +ve bacteria in this system, as in the previous assays. We then performed ex vivo stimulation of primary human whole-blood obtained from healthy donors via the TruCulture^®^ ex vivo whole-blood culture system. TruCulture^®^ tubes were loaded with 10^4^ particles of HK *Fusobacterium nucleatum* (Fn23726), *Fusobacterium nucleatum* (Fn25586), *Bifidobacterium breve* (Bb15700), or 1 ug/mL HMW Poly I:C. All tubes were incubated for 24 h (+/− 15 min) in a dry heat block at 37 ℃, without agitation. TruCulture^®^ tubes that were not loaded with HK bacteria or HMW Poly I:C served as a negative control. Following incubation, sera was harvested from all tubes as per manufacturer instructions and soluble immune mediators were measured via multi-analyte bead-based immune assay.

Consistent with what we have observed thus far, stratification and differentiation of HK Fn23726 and Fn25586 strains was also observed when using the TruCulture^®^ ex vivo whole-blood culture system, as seen in Figure 4. HK Fn23726 was superior to Fn25586 at stimulating primary human whole-blood and significantly upregulated in nearly all pro-inflammatory immune mediators, as seen in Figure 4A–E,G–I. Upregulation of TNFa was also seen in Figure 4L, though not statistically significant. Additionally, HKFn23726 was superior to Fn25586 in upregulating anti-inflammatory IL-10 in Figure 4J, though not statistically significant. Only two pro-inflammatory immune mediators, IL-12 (p70) and IP-10, were upregulated after stimulation of primary human whole-blood with HK Fn25586, compared to Fn23726, as seen in Figure 4F,K, respectively. HK Fn23726 also appears superior to HK Bb15700 at stimulating primary human whole-blood, as seen in Figure 4A–I,K, where these pro-inflammatory immune mediators are all significantly upregulated. Even amongst immune mediators in which HK Fn23726 is not significantly upregulated, levels of these immune mediators are trending higher than Bb15700, as seen with IL-12 (p40), IL-10, and TNFa, Figure 4F,J,L, respectively.

The TruCulture^®^ ex vivo immuno-modulatory signatures of Figure 4 overlap nicely, though not exactly, with primary human MoDC in vitro immuno-modulatory signatures observed in Figure 1 and Figure 3 and cell surface co-stimulation/maturation marker expression patterns in Figure 2. As with our primary human MoDC in vitro system, we see that primary human whole-blood stimulated with HK bacteria via the TruCulture^®^ ex vivo whole-blood culture system is also able to stratify and differentiate strains of HK bacteria with high confidence. The TruCulture^®^ ex vivo whole-blood culture system represents a more physiologically relevant environment and potentially enables faster screening of immuno-modulatory bacteria.

## 4. Discussion

Microbiome-based therapeutics range from the use of fecal microbiome transplant (FMT) [35,36], individual live commensal bacteria [37], consortia of previously well-defined bacteria [38] or commensal-based entities (secreted and encoded proteins/peptides/metabolites) [39,40]. However, the therapeutic potential use of killed/inactivated commensals has been incompletely evaluated. Despite many exciting breakthroughs in the pre-clinical space, microbiome-based modulators are yet to demonstrate superior clinical outcomes. While the field of microbiome research continues to evolve from pre-clinical animal model-based mechanistic research into a selection of lead candidates (bacteria and/or bacterial products) that can impact clinical outcomes, it is imperative to identify molecular mechanisms underlying microbiome-based modulation of the host. We believe that a major knowledge gap in identifying commensals of therapeutic potential in the human clinical setting is the lack of sensitive/predictive in vitro assays using primary human immune cells. This may largely be attributed to the complications associated with co-culturing anaerobic gut commensal bacteria with primary host immune cells that require aerobic growth conditions. Additionally, current in vitro methods of screening compounds, commensal bacteria, and others for potential immuno-modulation are hampered by the availability of sensitive, reliable cell lines and assays for detection of such potential. Here, we have shown that a primary human Monocyte-derived Dendritic Cell-based in vitro culture system may be uniquely well suited for this line of investigation.

To this end, we have demonstrated that primary human MoDC are capable of stratifying and differentiating different genera, species, and strains of commensal bacteria. This stratification and differentiation are based on their ability to modulate host immunity in an in vitro culture system. Subtle differences in the different strains of commensal bacteria used herein lead to different immuno-modulation of primary human MoDC. This immuno-modulation is evidenced by the upregulation of key cell surface co-stimulation/maturation markers including CD80, CD83, CD86, CD197 (CCR7), and HLA-DR. These cell surface markers all play key roles in the generation of an appropriate host immune response. CD80 and CD86 are essential for co-stimulation of T cells, whereas CD83 is a marker of DC maturation and important in antigen presentation, along with HLA-DR [41,42]. CD197 (CCR7), though not as strikingly upregulated, and its ligands link innate and adaptive immunity by affecting interactions between T cells and DC [43]. Additionally, the increased production of pro-inflammatory cytokines such as IL-1b, IL-6, IFNa2, and TNFa, amongst others, is critical to the development of the host response to foreign microorganisms, the vaccine-based immune response, and the response to cancer immunotherapy [44,45]. Furthermore, using the TruCulture^®^ ex vivo whole-blood culture system, we also see increased production of key pro-inflammatory cytokines, such as G-CSF, IL-8, and MIP-1a, amongst others. This, together with the consistency and reproducibility amongst donors in the cytokines measured, provides greater confidence in our in vitro findings since this is a more biologically relevant, translational ex vivo system comprised of multiple human immune cell subsets, immuno-modulatory cytokines, and other factors.

We believe the assays and read-outs described in this work offer a significant advantage over the use of traditional microbiome-based screening assays that have been published in the past. This advantage is illustrated by the critical finding that primary human MoDC are a sensitive cell type capable of differentiating between two different strains from the same species of bacteria, without the need for co-culturing with additional cell types. This ability to delineate strains based on their differences in immuno-modulation of primary human MoDC is crucial and should be a key criterion in the selection of lead candidates for evaluation in the clinic. For example, while differences in *Fusobacterium nucleatum* strains were consistently detected in this culture system, we acknowledge the growing body of literature implicating *Fusobacterium* in exacerbating oncogenic tumor activity [31,32]. Therefore, different species of *Fusobacterium* may be worth investigating using this in vitro culture system. Furthermore, follow-up studies are warranted to validate strong immuno-modulators observed in this study, such as *Fusobacterium nucleatum* (Fn23726), in an in vivo setting, including the employment of murine and non-human primate models. Another interesting observation from our results is the donor-to-donor variability of cell surface co-stimulation/maturation markers observed when primary human MoDC were stimulated with *Bifidobacterium breve* (Bb15700). *Bifidobacterium breve* and other *Bifidobacterium* species have been implicated in inducing beneficial immuno-modulatory effects in in vitro experiments, in vivo pre-clinical studies, and observation clinical studies [33,34,46,47,48]. As such, the use of the in vitro primary human immune cell-based assay described in this study, may be able to build upon these data and further our understanding of the different immuno-modulatory characteristics of different bacterial species.

Despite the donor-to-donor variability inherent in the use of primary human cells, we believe the methods described herein are robust and represent a consistent way of evaluating potential commensal bacteria-based immune modulation. We propose using this combination of primary human MoDC in vitro culture system and TruCulture^®^ ex vivo whole-blood culture system as an expeditious screening assay in the search for microbiome-based therapeutics. Such detailed understanding is critical to the delineation and selection of the optimal commensal bacterial species and/or strains for use in the clinic as potential immuno-modulators in the context of allergy, auto-immunity, immuno-oncology, or as vaccine delivery vehicles/adjuvants.

## 5. Conclusions

Here, we describe the use of primary human MoDC and whole-blood based assays and read-outs that offer a significant advantage over the use of traditional microbiome-based screening assays. By the strategic selection and usage of representative Gram-positive and Gram-negative bacterial strains, we demonstrate that use of heat-killed bacteria may offer significant advantages over the use of live bacteria. In addition, we reveal that alteration in specific cytokines and co-stimulatory/maturation markers in primary human MoDC-based assays are sensitive enough to differentiate (i) various strains of commensal bacteria as well as (ii) between two different strains from the same species of bacteria. Furthermore, we validated our MoDC based findings using a clinically used human whole-blood based TruCulture^®^ assay system. As such, we propose using this combination of primary human MoDC in vitro culture system and TruCulture^®^ ex vivo whole-blood culture system as an expeditious screening assay in the search for reliable microbiome-based therapeutics.

## Figures and Tables

**Table 1:** Bacterial Genus, Species, and Strains Investigated Using Primary Human MoDC. Seven anaerobic bacteria, one facultative-anaerobic bacterium, and one aerobic bacterium (six Gram negative [Gram -ve] and three Gram positive [Gram +ve] were selected to validate this primary human MoDC in vitro culture system.

Bacteria	Strain Number	Source	Gram	Order	Aerobe/Anaerobe
*Akkermansia muciniphila* (Am)	BAA-835	ATCC	Gram -ve	Verrucomicrobiales	Anaerobe
*Fusobacterium nucleatum* (Fn)	23726	ATCC	Gram -ve	Fusobacteriales	Anaerobe
*Fusobacterium nucleatum* (Fn)	25586	ATCC	Gram -ve	Fusobacteriales	Anaerobe
*Bacteroides fragilis* (Bf)	25285	ATCC	Gram -ve	Bacteroidales	Anaerobe
*Bacteroides fragilis* (Bf)	43858	ATCC	Gram -ve	Bacteroidales	Anaerobe
*Escherichia coli K-12* (K12)	10798	ATCC	Gram -ve	Enterobacterales	Aerobe
*Bifidobacterium breve* (Bb)	15700	ATCC	Gram +ve	Bifidobacteriales	Anaerobe
*Faecalibacterium prausnitzii* (Fp)	27766	ATCC	Gram +ve	Clostridiales	Anaerobe
*Enterococcus hirae* (Eh)	8043	ATCC	Gram +ve	Lactobacillales	Facultative Anaerobe

## Supplementary Materials

The following are available online at https://www.mdpi.com/2076-393X/9/3/225/s1, Figure S1: Primary Human MoDC Show Increased ATP Release When Stimulated with Live Bacteria vs. Heat-Killed Bacteria, Figure S2: Representative Gating Strategy Applied to All Primary Human MoDC Donors, Figure S3: Stimulation of Primary Human MoDC Shows Upregulation of Activation Markers With Poly I:C and LPS Controls, Table S1: Statistical Comparison of Mean Fluorescence Intensity Across the Various Bacterial Strains Tested Against Negative Control (DPBS) and Positive Control (Poly I:C), Table S2: Statistical Comparison of Soluble Mediators Across the Various Bacterial Strains Tested Against Negative Control (DPBS) and Positive Control (Poly I:C).
