# Supplementary material for: Primary Human Dendritic Cells and Whole-Blood Based Assays to Evaluate Immuno-Modulatory Properties of Heat-Killed Commensal Bacteria
Source: Vaccines (Basel). 2021 Mar 5;9(3):225. doi: 10.3390/vaccines9030225 (PMC8001086; doi:10.3390/vaccines9030225)
Supplement: Supplementary file 1 [file vaccines-09-00225-s001.pdf]

Supplementary Figures

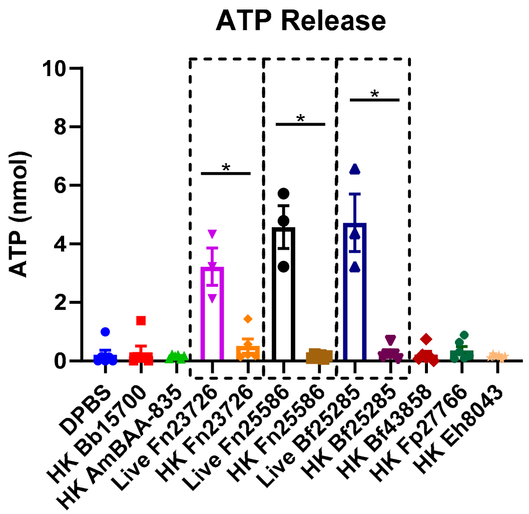

Figure S1: Primary Human MoDC Show Increased ATP Release When Stimulated with Live Bacteria vs. Heat-Killed Bacteria. Cytotoxicity of live and heat-killed bacteria was analyzed in primary human MoDC, as measured by extracelllular ATP release. The mean + standard error of the mean (SEM) is indicated. To compare two groups of data, an unpaired parametric *t*-test with Welch's correction was used. N = 3-6 independent donors. (**p* <0.05)

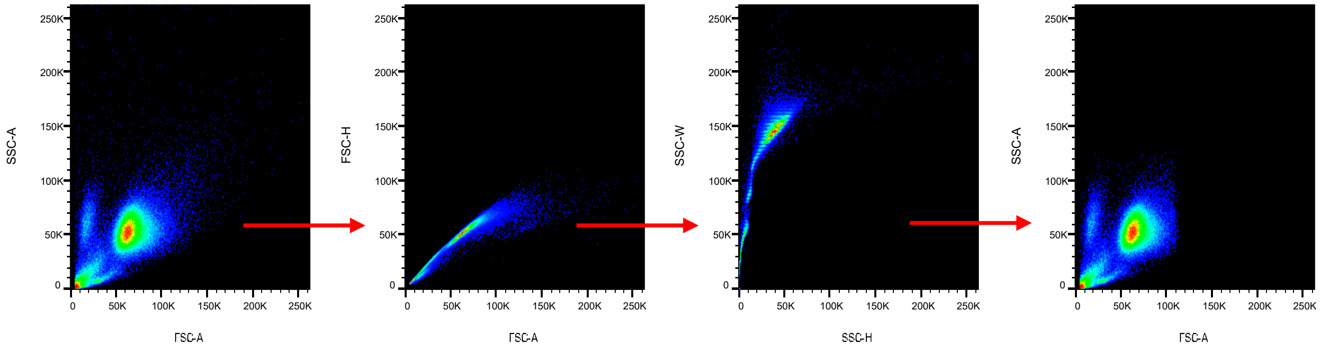

Figure S2: Representative Flow Cytometry Gating Strategy Applied to All Primary Human MoDC Donors. Primary human MoDC were gated using FlowJo V10.4.2 software prior to analysis of mean fluorescence intensity (MFI) for cell surface activation markers CD80, CD83, CD86, CD197 (CCR7), and HLA-DR. All primary human MoDC donors were subjected to the same gating strategy.

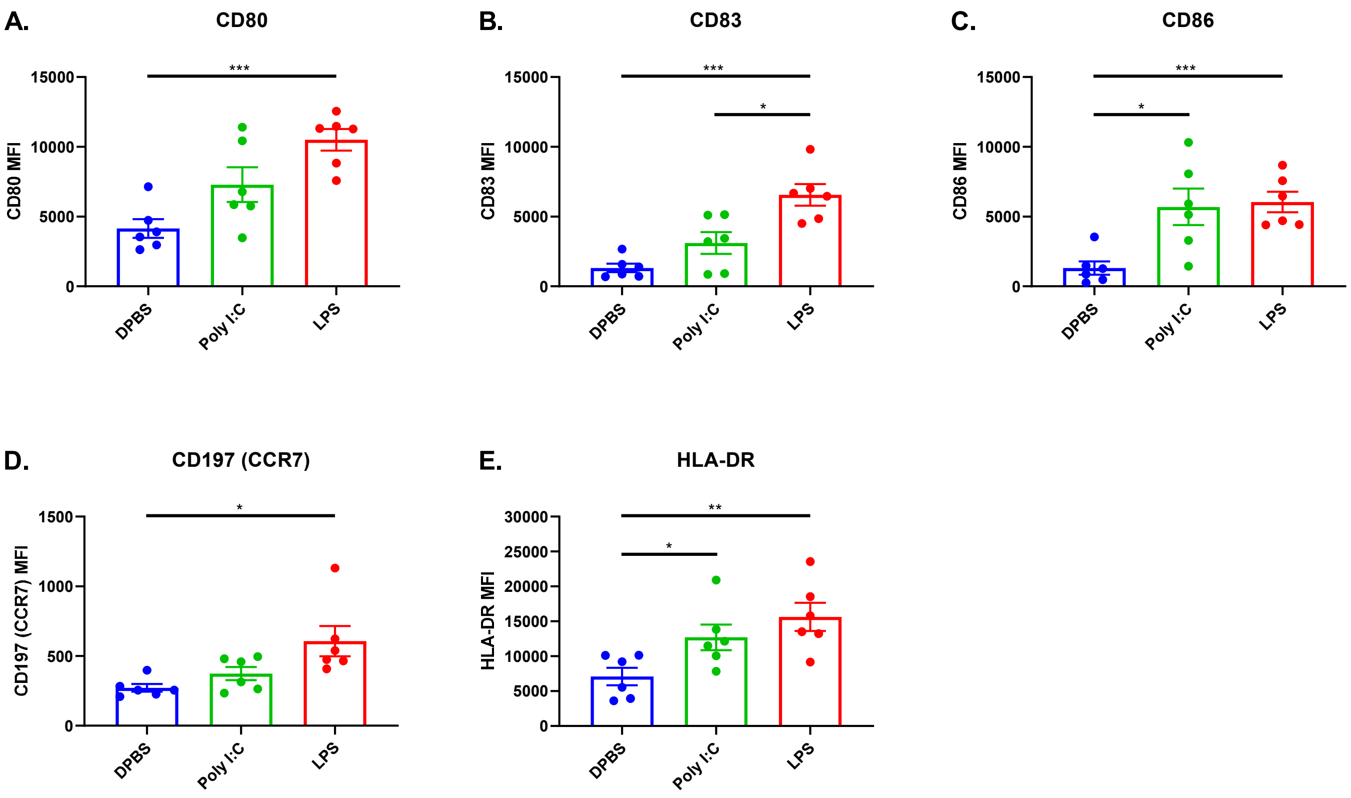

Figure S3: Stimulation of Primary Human MoDC Shows Upregulation of Activation Markers With Poly I:C and LPS Controls. Primary human MoDC were analyzed for activation, as measured by the upregulation of cell surface expression of CD80, CD83, CD86, CD197 (CCR7), and MHCII in heat-killed bacteria-stimulated cultures. The mean fluroescence intensity (MFI) of CD80 (A), CD83 (B), CD86 (C), CD197 (CCR7) [D], and HLA-DR (E) are indicated. To compare two groups of data, an unpaired parametric *t*-test with Welch's correction was used. N = 6 independent donors. (*p <0.05, **p <0.01, ***p <0.001)

Table S1: Statistical Comparison of Mean Fluorescence Intensity Across the Various Bacterial Strains Tested Against Negative Control (DPBS) and Positive Control (Poly I:C). Primary human MoDC were analyzed for activation, as measured by upregulation of cell surface expression of CD80, CD83, CD86, CD197 (CCR7), or MHCII in heat-killed bacteria-stimulated cultures. Statictical comparison of the mean fluorescence intensity (MFI) of CD80 (A), CD83 (B), CD86 (C), CD197 (CCR7) [D], and HLA-DR (E) of DPBS- and Poly I:C-stimulated primary human MoDC to various bacterial strains are indicated. To compare two groups of data, an unpaired parametric *t*-test with Welch's correction was used. N = 6 independent donors. (*p <0.05, **p <0.01).

	DPBS	Poly I:C	K12	Bb15700	AmBAA-835	Fn23726	Fn25586	Bf25285	Bf43858	Fp27766	Eh8043
**(A) CD80**											
**DBPS**	—	0.0580	0.0988	* 0.0224	0.3990	** 0.0032	0.09519	0.1905	** 0.0015	0.7256	0.1760
**Poly I:C**	0.0580	—	0.9890	0.2812	0.2124	0.0562	0.0547	0.3079	0.3525	0.0936	0.4249
**(B) CD83**											
**DBPS**	—	0.0728	0.0590	0.0624	0.4093	* 0.0157	0.9537	0.1991	** 0.0098	0.8825	0.1266
**Poly I:C**	0.0728	—	0.7751	0.2712	0.2857	0.0882	0.0708	0.2269	0.1302	0.8480	0.6722
**(C) CD86**											
**DBPS**	—	* 0.0185	0.0806	** 0.0092	0.1938	** 0.0032	0.9245	0.2460	** 0.0064	0.5212	0.0950
**Poly I:C**	* 0.0185	—	0.4450	0.6998	0.2006	0.5915	*0.0197	0.0540	0.7043	* 0.0324	0.4543
**(D) CD197 (CCR7)**											
**DBPS**	—	0.0988	0.0632	0.1461	0.7063	* 0.0399	0.8274	0.5660	* 0.0322	0.9585	0.4544
**Poly I:C**	0.0988	—	0.5261	0.3149	0.1673	0.0683	0.0775	0.1947	0.0380	0.1100	0.3025
**(E) HLA-DR**											
**DBPS**	—	* 0.0330	0.0871	* 0.0181	0.3287	** 0.0049	0.9872	0.3376	** 0.0033	0.6632	0.1183
**Poly I:C**	* 0.0330	—	0.8130	0.4670	0.2170	0.2351	* 0.0314	0.1706	0.5303	0.0622	0.4116

Table S2: Statistical Comparison of Soluble Mediators Across the Various Bacterial Strains Tested Against Negative Control (DPBS) and Positive Control (Poly I:C). Primary human MoDC were analyzed for activation, as measured by upregulation of secreted, soluble mediators in cell culture supernatant. Statictical comparison of the mean + standard error of the mean (SEM) of G-CSF (A), IFNα2 (B), IL-1β (C), IL-6 [D], IL-12 (p40) [E], IL-12 (p70) [F], MIP-1α (G), MIP-1β (H), IL-8 (I), IL-10 (J), IP-10 (K), and TNFα (L) of DPBS- and Poly I:C-stimulated primary human MoDC to various bacterial strains are indicated. To compare two groups of data, an unpaired parametric *t*-test with Welch's correction was used. N = 6 independent donors. (*p <0.05, **p <0.01, ***p <0.001, ****p <0.0001).

	DPBS	Poly I:C	K12	Bb15700	AmBAA-835	Fn23726	Fn25586	Bf25285	Bf43858	Fp27766	Eh8043

**(A) G-CSF**											
**DBPS**	—	0.0887	0.078	0.1724	0.5878	0.1181	0.8909	0.1734	* 0.0343	0.6978	0.0710
**Poly I:C**	0.0887	—	0.1134	0.2401	0.1155	0.1306	0.0936	0.1753	0.1386	0.1034	0.5116
**(B) IFNα2**											
**DBPS**	—	0.1899	**** <0.0001	0.1473	0.7248	*** 0.0003	0.8855	0.4957	** 0.0084	0.9677	* 0.0411
**Poly I:C**	0.1899	—	0.6232	0.5317	0.2046	0.9613	0.1948	0.2150	0.4895	0.1883	0.6172
**(C) IL-1β**											
**DBPS**	—	0.2895	0.1200	0.1051	0.5001	* 0.0364	0.9309	0.3489	0.1810	0.8042	0.1535
**Poly I:C**	0.2895	—	0.1688	0.5486	0.2975	* 0.0408	0.2904	0.4393	0.3712	0.2870	0.8261
**(D) IL-6**											
**DBPS**	—	0.1066	0.2019	0.1940	0.2919	0.2240	0.4906	0.3372	0.0574	0.3937	0.0727
**Poly I:C**	0.1066	—	0.2092	0.1059	0.1118	0.2302	0.1096	0.8718	0.0657	0.1075	0.1009
**(E) IL-12 (p40)**											
**DBPS**	—	0.3271	0.0778	0.2157	0.8398	0.2601	0.6595	0.2759	* 0.0136	0.9358	* 0.0262
**Poly I:C**	0.3271	—	0.2945	0.3964	0.3275	0.2658	0.3265	0.3306	0.6345	0.3270	0.5479
**(F) IL-12 (p70)**											
**DBPS**	—	0.2333	*0.0370	0.1381	0.5936	0.1336	0.3121	0.2554	0.0599	0.6630	* 0.0165
**Poly I:C**	0.2333	—	0.2768	0.4590	0.2339	0.1405	0.2345	0.2728	0.4502	0.2337	0.1782
**(G) MIP-1α**											
**DBPS**	—	0.2815	0.0522	0.3940	0.4684	0.2919	0.5180	0.2311	* 0.0367	0.8285	0.2957
**Poly I:C**	0.2815	—	0.9335	0.4267	0.2820	0.4783	0.2820	0.2852	0.6748	0.2816	0.8786
**(H) MIP-1β**											
**DBPS**	—	0.2645	* 0.0227	0.3950	0.4030	0.1140	0.5267	0.3383	* 0.0210	0.8030	0.1649
**Poly I:C**	0.2645	—	0.4617	0.3012	0.2653	0.1975	0.2647	0.2740	0.5392	0.2646	0.7291
**(I) IL-8**											
**DBPS**	—	0.3547	*** 0.0006	0.3928	0.2738	*** 0.0008	0.3595	0.3026	** 0.0039	0.9149	0.1412
**Poly I:C**	0.3547	—	** 0.0011	0.5286	0.3888	** 0.0039	0.3734	0.9265	** 0.0075	0.3557	0.8562
**(J) IL-10**											
**DBPS**	—	0.3377	0.1210	0.2856	0.9690	0.1754	0.8989	0.3728	0.1719	0.8688	0.2106
**Poly I:C**	0.3377	—	0.1666	0.3016	0.3371	0.1768	0.3358	0.3691	0.2864	0.3404	0.8117
**(K) IP-10**											
**DBPS**	—	0.1524	0.0606	0.3547	0.3819	0.1672	0.4039	0.1349	0.0749	0.5928	0.1304
**Poly I:C**	0.1524	—	0.1884	0.8068	0.1525	0.7595	0.1524	0.1527	0.1617	0.1524	0.2008
**(L) TNFα**											
**DBPS**	—	0.2654	** 0.0094	0.1195	0.2716	*** 0.0002	0.3544	0.2243	0.0731	0.5135	* 0.0270
**Poly I:C**	0.2654	—	0.1141	0.6471	0.2689	* 0.0101	0.2686	0.2706	0.7941	0.2659	0.7644
